# Primary Tumor Histology Affects Oncological Outcomes Independently of the Anatomical Extent of Disease in Colorectal Liver Metastasis

**DOI:** 10.31662/jmaj.2018-0004

**Published:** 2020-07-07

**Authors:** Hideki Ueno, Tsuyoshi Konishi, Yuichi Ishikawa, Hideyuki Shimazaki, Masashi Ueno, Suefumi Aosasa, Akio Saiura, Eiji Shinto, Yoshiki Kajiwara, Satsuki Mochizuki, Takahiro Nakamura, Junji Yamamoto

**Affiliations:** 1 Department of Surgery, National Defense Medical College, Saitama, Japan; 2 Gastroenterology Center, Cancer Institute Hospital, Tokyo, Japan; 3 Division of Pathology, Cancer Institute Hospital, Tokyo, Japan; 4 Department of Laboratory Medicine, National Defense Medical College, Saitama, Japan; 5 Laboratory for Mathematics, National Defense Medical College, Tokorozawa, Japan; 6 Laboratory for Statistical Analysis, Center for Genomic Medicine, RIKEN, Tokyo, Japan

**Keywords:** colorectal liver metastasis, desmoplastic reaction, epithelial-mesenchymal transition (EMT), hepatectomy, poorly differentiated cluster

## Abstract

**Introduction:**

Growing evidence indicates the prognostic importance of the crosstalk between cancer cells and stroma through the induction of epithelial-mesenchymal transition (EMT). This study aimed to clarify the prognostic value of evaluating primary tumor histology with the anatomical extent of disease in patients with colorectal liver metastasis (CRLM).

**Methods:**

Prognostic analyses were performed in 411 CRLM patients who underwent hepatectomy at two institutions. Tumors were graded into one of three histological categories based on integrated assessment of EMT-associated histology (Histology^EMT^) in primary tumors, i.e., poorly differentiated clusters (PDCs) and desmoplastic reaction (DR).

**Results:**

A prognostic grouping system for the anatomical extent of disease (N stage, liver metastasis number and size, and extrahepatic disease; Grade^anatomical^) stratified patients into three groups with different five-year relapse-free survival (RFS) rates after hepatectomy: A, 31% (191 patients); B, 15% (124 patients); and C, 6% (96 patients; *P* < 0.0001). Histology^EMT^ (A, G1 PDC and mature-type DR; C, G3 PDC and immature-type DR; and B, others) identified 49, 120, and 242 patients with 46%, 5%, and 22% five-year RFS, respectively (*P* < 0.0001). Among prognostic factors, the Akaike information criterion was most favorable in Grade^anatomical^, followed by Histology^EMT^. Multivariate analysis demonstrated that these two factors independently impacted RFS; two-year RFS after hepatectomy in different patient groups had a wide range (10%-76%).

**Conclusions:**

Histological assessment of dedifferentiation and the stromal environment of primary tumors contributed to effective risk stratification of early relapse after hepatectomy, which could be useful to determine clinical management for CRLM patients.

## Introduction

CRC patients with resectable liver metastases comprise a prognostically heterogeneous population that requires treatment on a case-to-case basis, especially in accordance with the accurate evaluation of recurrence risk within a short period after hepatectomy. Since the 1990s, several prognostic prediction systems have been proposed for CRC liver metastasis patients undergoing hepatectomy ^(1), (2), (3), (4)^. Most of these systems are intended to determine the characteristics of patients suitable for hepatectomy. These comprise conventional prognostic markers of the anatomical extent of disease, such as liver metastasis number ^(1), (2), (3), (4)^ and size ^(1), (2), (4)^, resection margin ^(1)^, extrahepatic disease ^(4)^, disease-free interval ^(1), (2), (3)^, primary tumor nodal status ^(1), (2), (4)^, and serum carcinoembryonic antigen (CEA) levels ^(1), (2)^. However, their broad application currently has limited value in patient stratification for clinical management ^(5)^, principally owing to the inability to accurately stratify patients according to the prognostic outcome.

Recent accumulated knowledge in cancer biology research highlights the importance of the molecular mechanism of epithelial-mesenchymal transition (EMT), a means by which transformed epithelial cells can acquire the ability to invade and disseminate ^(6)^. Under the EMT program, loss of differentiation is induced at the tumor-host interface in CRC, which enables cellular detachment, dissemination, and eventually, metastasis ^(7), (8)^. In the cancer microenvironment, cancer-associated fibroblasts (CAFs) play a central role in mediating the EMT program ^(8), (9)^, thereby facilitating the progression of cancer cells toward a dedifferentiated state. One of the most promising methods for characterizing dedifferentiation to assess the migratory phenotype of a tumor is to quantify poorly differentiated clusters (PDCs) ^(10), (11)^. Additionally, desmoplastic reaction (DR), histological consequences of extracellular matrix remodeling generated by CAFs, can be morphologically categorized on the basis of the presence of specific types of fibrous cancer stroma ^(12)^.

**Figure 1.**
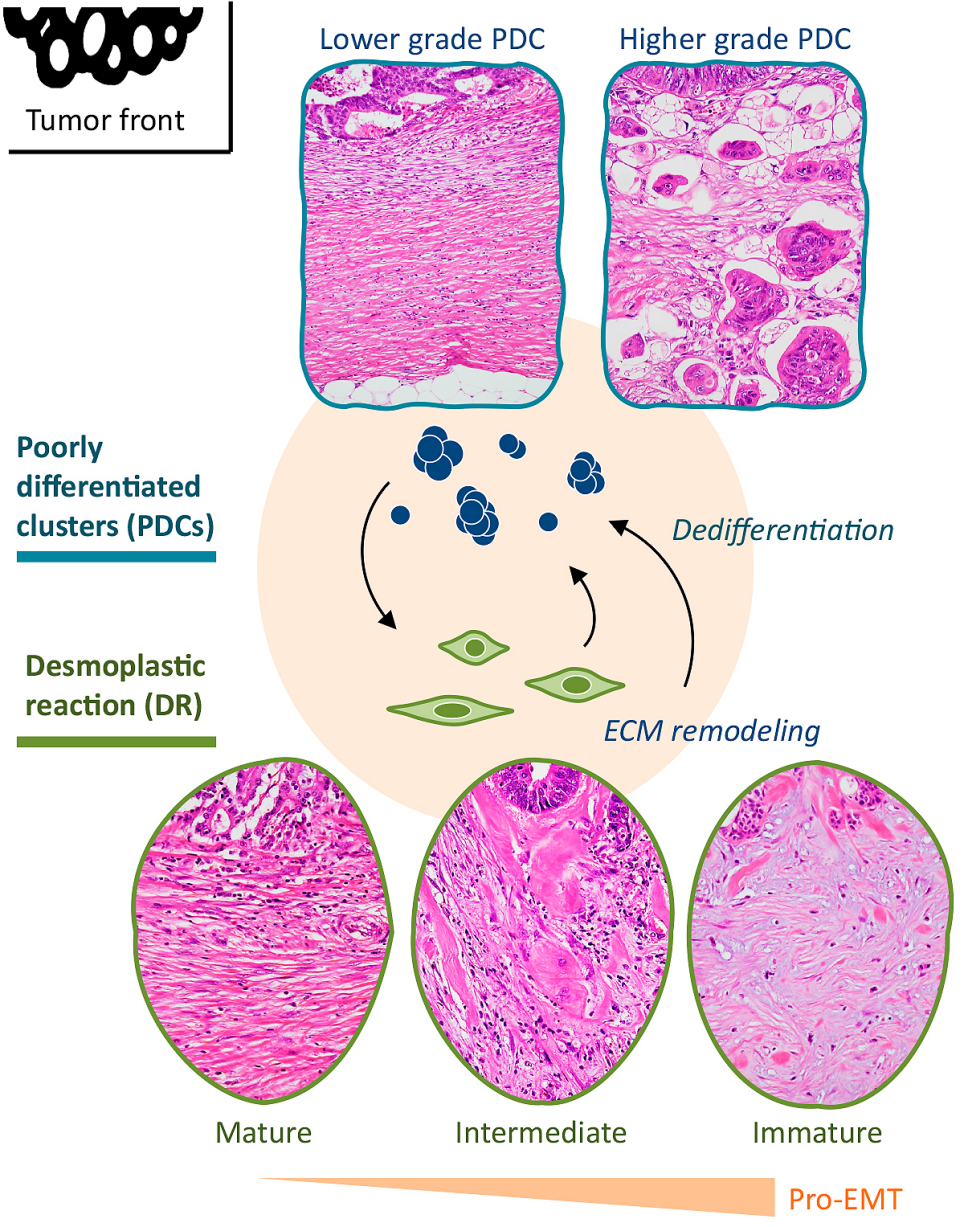
Scheme of histological heterogeneity at the primary tumor front in terms of the association between desmoplastic patterns and dedifferentiation. CAFs are being increasingly recognized as central players in modulating the metastatic capacity of most cancers. Directly and indirectly, the oncological outcome of tumors is determined during the process of DR through the formation of the extracellular matrix, which causes EMT signals to induce tumor cells into a dedifferentiated phenotype. In the present study, comprehensive assessment of PDCs and the DR pattern (Histology^EMT^) was used as an index to assess the individual tumor microenvironments associated with the EMT status. PDCs are cancer cell clusters composed of ≥ 5 cancer cells that lack gland formation and are located in the stroma. PDCs were graded as 1, 2, or 3 using a hot-spot method. According to the existence of hyalinized collagens and myxoid stroma, which are distinctive fibrotic stromal components that exclusively appear at the desmoplastic front and are thought to be morphological results of activated CAF function, DR was classified into three patterns: mature (neither hyalinized collagens nor myxoid stroma), intermediate (fibrotic stroma with hyalinized broad bundles), or immature (fibrotic stroma with myxoid changes). PDCs, poorly differentiated clusters; CAFs, cancer-associated fibroblasts; ECM, extracellular matrix; EMT, epithelial-mesenchymal transition.

Recently, we proposed a histological categorization of EMT (Histology^EMT^) by the integrated assessment of PDC and DR on the premise that Histology^EMT^ represents the potential of induction of EMT in CRC (Figure 1) ^(13)^. Our previous study indicated that this categorization greatly influences oncological results not only in patients with curatively resected CRC but also in stage IV patients with unresectable distant metastasis ^(13)^. The aims of the present study were to examine the prognostic value of Histology^EMT^ in a multi-institutional patient dataset that underwent curative hepatectomy for CRC metastases and to clarify whether comprehensive assessment of the histological expression of CRC metastatic potential in primary tumors and the anatomical extent of disease were useful for evaluating the survival outcome, particularly the risk of early relapse after hepatectomy.

## Materials and Methods

### Patients

The study included CRC patients (n = 411) who had undergone potentially curative surgery for their primary tumors and synchronous/metachronous liver metastases at two independent institutions: the National Defense Medical College Hospital (1984-2010) and the Cancer Institute Hospital (1995-2007). The average age of the patients was 61.4 years (range, 28-91 years), with 271 males and 140 females. Of these, 245 patients had colon cancer and 166 had rectal cancer. The average and median follow-up period after hepatectomy for survivors was 61 and 48 months, respectively (range, 11-188 months). This study protocol was approved by the Ethics Committee of the National Defense Medical College (No.2954) and the Cancer Institute Hospital (No. 2010-1056).

### Primary tumor histology

One of the authors (HU) pathologically reviewed primary tumors to evaluate PDCs and DR with no prior knowledge of the patients' clinical outcomes. Hematoxylin-eosin (HE)-stained glass slides prepared from a single longitudinal section of the whole tumor, including its deepest part, were used to determine the grade of PDCs and pattern of DR.

#### PDCs

PDCs were defined as cancer cell clusters comprising ≥ 5 cancer cells infiltrating the stroma and lacking gland formation ^(10), (11)^. After selecting one field where PDCs were the most intensive, the number of clusters was counted under a 20× objective lens, and the grade was determined on the basis of the number of clusters present. Tumors with < 5, 5-9 and ≥10 clusters were classified as G1, G2, and G3, respectively.

#### DR

The DR pattern was histologically classified into one of three categories (mature, intermediate, and immature) on the basis of the existence of keloid-like collagen and myxoid stroma in the reactive fibrous zone at the advancing edge of the tumor ^(12)^. Keloid-like collagen consists of broad bundles of hypocellular collagen with brightly eosinophilic hyalinization, typically observed in a keloid. Myxoid stroma can be defined as an amorphous stromal substance composed of an amphophilic or slightly basophilic material that is usually intermingled with randomly oriented keloid-like collagen.

DR was regarded as a mature pattern when fibrotic stroma did not contain keloid-like collagen or myxoid stroma and consisted of fine mature collagen fibers stratified into multiple layers. When keloid-like collagen was intermingled with mature stroma, the fibrotic stroma was designated as undergoing intermediate maturation. Stroma with myxoid changes was regarded as immature stroma. In each case, stroma was classified according to the most immature stromal area.

#### Categorization criteria for Histology^EMT^

**Figure 2.**
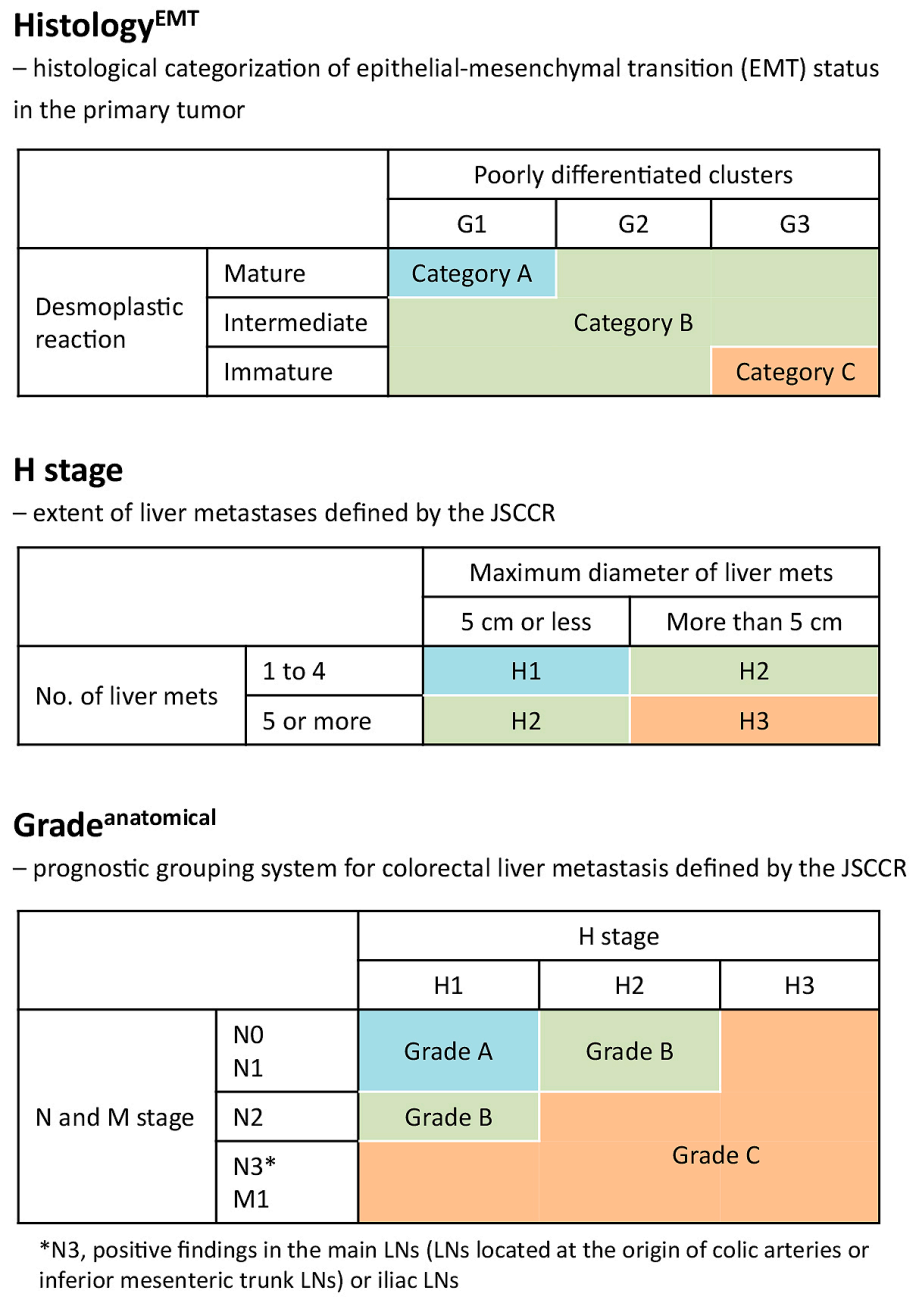
Categorization systems of Histology^EMT^, H stage, and Grade^anatomical^. JSCCR, Japanese Society for Cancer of the Colon and Rectum.

Using PDC and DR as components, a three-tiered categorization system (Histology^EMT^) was established as the model to estimate the potential of EMT of a tumor (Figure 2) ^(13)^. Category A included tumors with both G1 PDC and mature-type stroma; category C included tumors with both G3 PDC and immature-type stroma; and category B indicated tumors with other histological types.

### Anatomical extent of disease

The T and N stages in the primary tumor, liver metastasis number and size, index for the extent of liver metastases defined by the Japanese Society for Cancer of the Colon and Rectum (JSCCR; H stage) ^(14)^, and extrahepatic disease were analyzed as parameters for the anatomical extent of disease. We evaluated prognostic value of the number and size of liver metastasis with cutoff values used to define H stage, i.e., five for the number and 5 cm for the size of metastasis, respectively. The prognostic grouping system defined by JSCCR (Grade^anatomical^) described below was used as a prognostic prediction model to comprehensively assess the anatomical extent of disease (Figure 2) ^(14)^.

#### H stage

The extent of liver metastasis was stratified as follows: H1, 1-4 metastatic tumors, all of which are ≤ 5 cm in maximum diameter; H3, ≥ 5 metastatic tumors, at least one of which is ≥5 cm in maximum diameter; and H2, other than H1 or H3 ^(14)^.

#### Grade^anatomical^

The three-tiered grading system of the anatomical extent of disease was determined on the basis of the primary tumor N stage, H stage, and extrahepatic metastases ^(14)^. In summary, the modified TNM staging system was used to determine the primary tumor N stage: N0, no lymph node (LN) metastasis; N1, 1-3 LN metastasis; N2, ≥4 LN metastasis; N3, positive findings in the main LNs (LNs located at the origin of each colic artery and inferior mesenteric trunk LNs) or iliac LNs ^(14)^. Patients without any extrahepatic metastases were classified as Grade^anatomical^ A or B: grade A denoted patients with N0/1 and H1 and grade B denoted patients with N2 and H1 or N0/1 and H2. Grade C denoted patients with extrahepatic metastasis or the following combinations of N and H stages: N0/1 and H3, N2 and H2/3, or N3 and any H stage.

### Statistical analyses

Survival rates were calculated using the Kaplan-Meier method, and comparisons were made using the log-rank test. After categorization, each clinical and pathological variable was entered into Cox’s proportional hazard regression analysis to determine the hazard ratio and 95% confidence interval for survival after hepatectomy and to compare the prognostic power of each factor using the Akaike information criterion (AIC) ^(15)^; the simplest, most effective model with the least information loss while predicting the outcome provides the lowest AIC value. The associations among prognostic factors and their associations with recurrence were analyzed using the chi-square test. The chi-square test was used to assess the differences in recurrence rates according to Histology^EMT^. Statistical analyses were conducted using SPSS Statistics 17.0 (SPSS, Inc., Chicago, IL, USA), Stata/SE 10 (StataCorp LP, College Station, TX, USA), and StatView ver.5.0 (SAS Institute, Inc., Cary, NC, USA).

## Results

### Correlation between PDCs and DR and distribution of Histology^EMT^ categories

There was a significant correlation between PDCs and DR (*P* < 0.0001), but multivariate analysis indicated an independent impact of PDCs and DR on both relapse-free survival (RFS;* P* = 0.0016 and 0.0066, respectively) and overall survival (OS;* P* = 0.0135 and 0.0022, respectively).

According to Histology^EMT^, primary tumors were classified as categories A, B, and C (49, 242, and 120 tumors, respectively). Histology^EMT^ was significantly associated with the T stage, N stage, tumor grade, venous invasion, metastasis timing, extrahepatic disease, and Grade*^anatomical^* (*P* ≤ 0.0001-0.001) and not with liver-associated factors (number and size as well as H stage).

### Prognostic impact of Histology^EMT^

**Figure 3.**
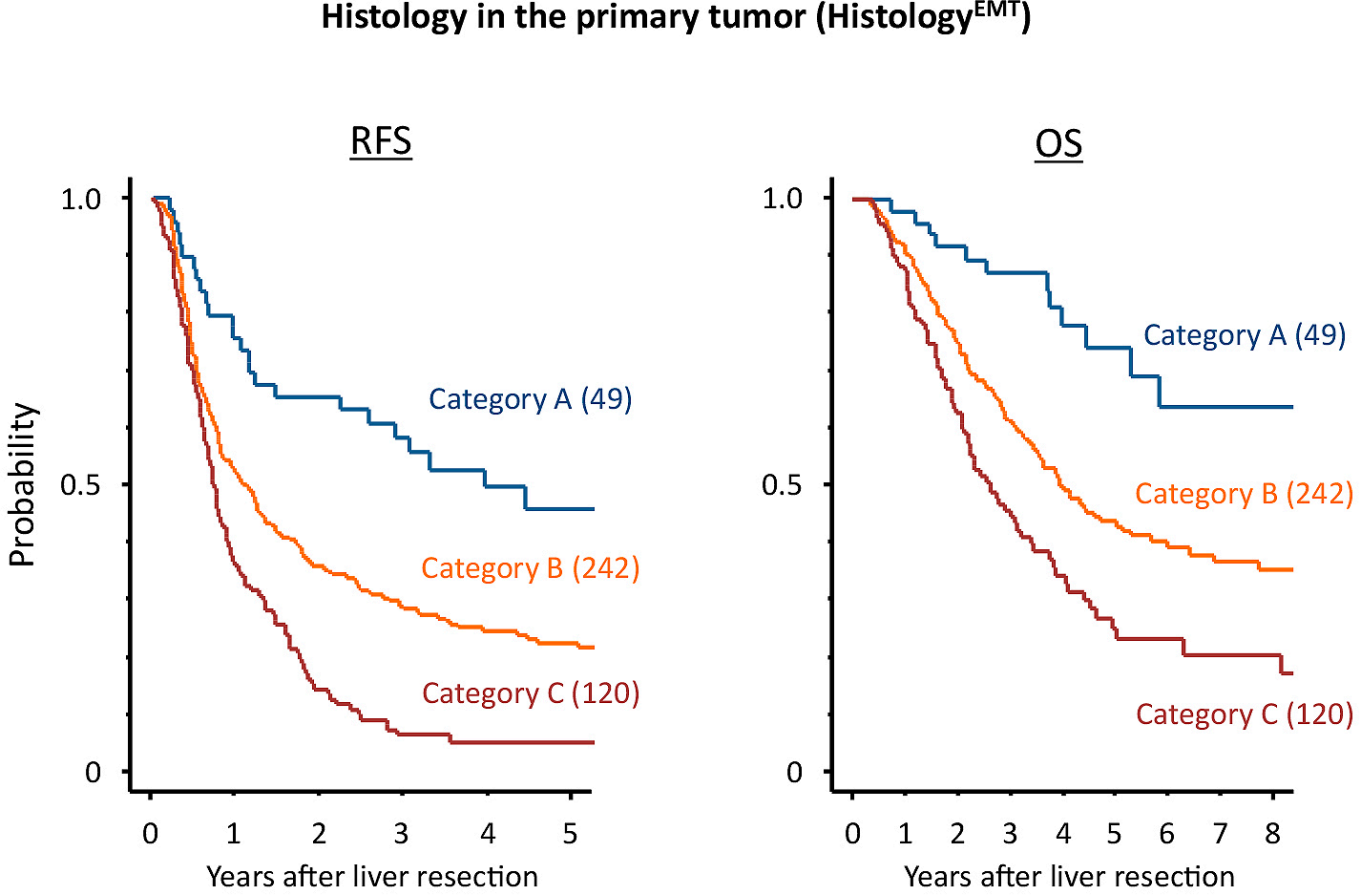
Survival estimates of patients undergoing hepatectomy for colorectal liver metastases according to primary tumor histology (Histology^EMT^) using the Kaplan-Meier method. Five-year RFS by Histology^EMT^: category A, 45.8%; category B, 22.4%; category C, 5.0% (*P* < 0.0001); Five-year OS by Histology^EMT^: category A, 74.1%; category B, 43.8%; category C, 25.1% (*P* < 0.0001). RFS, relapse-free survival; OS, overall survival. The numbers in parentheses denote the number of patients.

**Figure 4.**
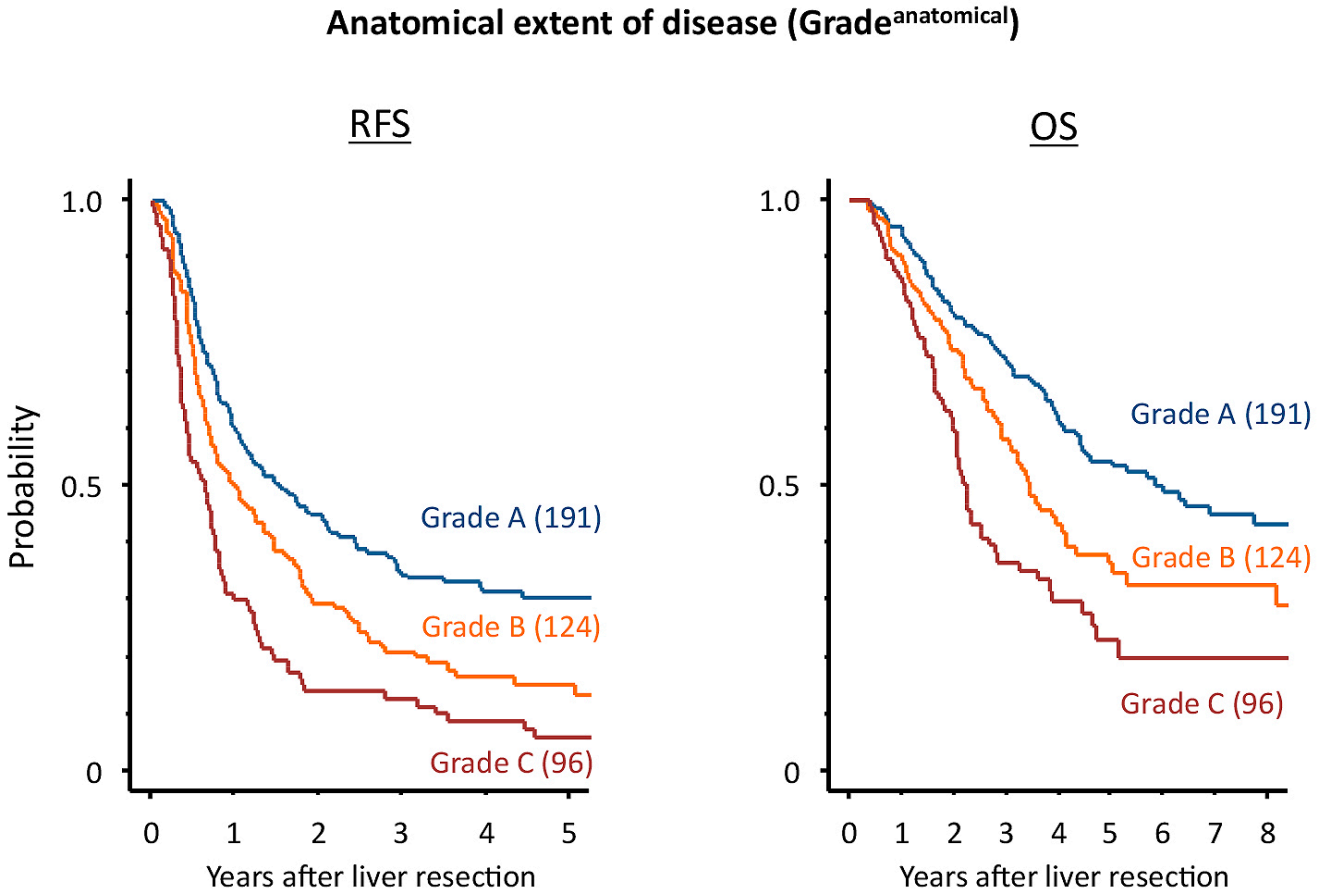
Survival estimates of patients undergoing hepatectomy for colorectal liver metastases according to the anatomical extent of disease (Grade^anatomical^) using the Kaplan-Meier method. Five-year RFS by Grade^anatomical^: grade A, 30.6%; grade B, 15.4%; grade C, 5.9% (*P* < 0.0001); Five-year OS by Grade^anatomical^: grade A, 54.4%; grade B, 36.5%; grade C, 23.1% (*P* < 0.0001). RFS, relapse-free survival; OS, overall survival. The numbers in parentheses denote the numbers of patients.

Prognostic factors associated with RFS after hepatectomy are shown in Table 1. According to Histology^EMT^, five-year RFS after hepatectomy for categories A, B, and C were 45.8%, 22.4%, and 5.0%, respectively (*P* < 0.0001). Among the prognostic factors examined, Histology^EMT^ had the second most favorable AIC (3423.0) following Grade^anatomical^ (3417.5) which stratified patients with different five-year RFS as 30.6% for grade A, 15.4% for grade B, and 5.9% for grade C (*P* < 0.0001). Figures 3 and 4 show the Kaplan-Meier estimates for RFS after hepatectomy in patients stratified into the three groups according to Histology^EMT^ and Grade^anatomical^, respectively.

**Table 1.**
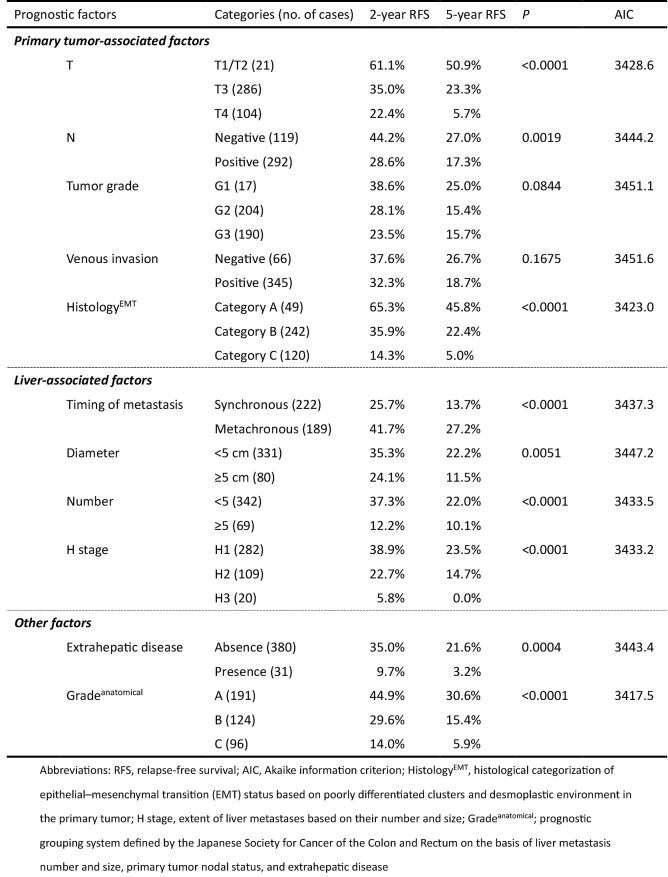
Prognostic Factors Associated with Relapse-Free Survival after Hepatectomy for Colorectal Liver Metastasis.

With regard to the recurrence pattern, Histology^EMT^ was significantly associated with hepatic, lung, peritoneal, and local recurrence (Table 2). The incidence of overall recurrence was the highest in category C (92.5%), followed by categories B (73.6%) and A (46.9%; P < 0.0001). A significant impact of Histology^EMT^ on the overall recurrence rate was observed in the subset analyses of institution cohort, metastasis timing, and primary tumor location.

**Table 2.**
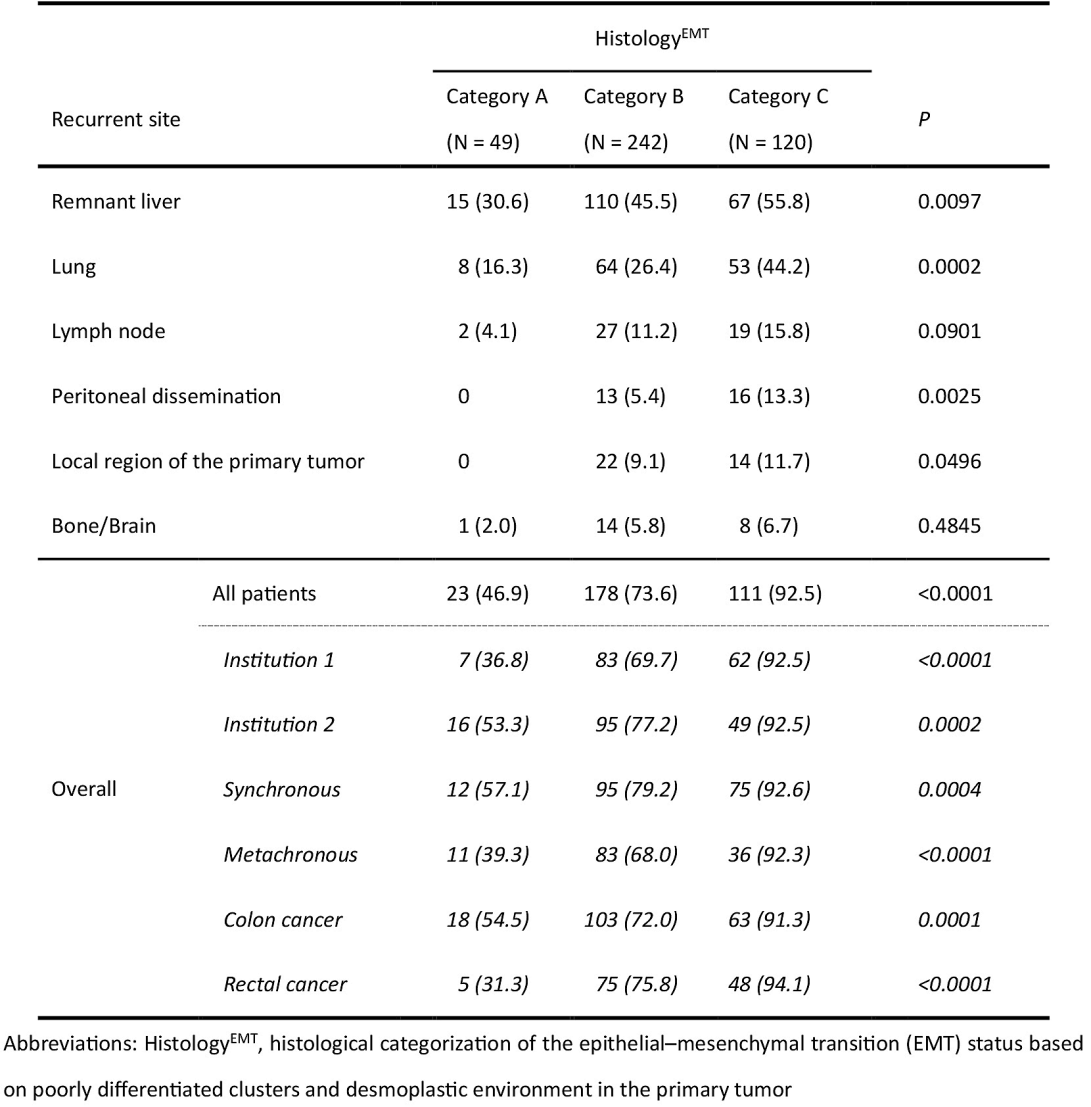
Impact of Histological Categorization of the EMT Status in the Primary Tumor (Histology^EMT^) on Recurrence Pattern after Hepatectomy.

### Multivariate analysis

In multivariate analyses for RFS, parameters associated with the anatomical extent of disease were analyzed under three different conditions (Table 3): set 1, all factors entered the multivariate analysis individually; set 2, H stage entered the multivariate analysis instead of liver metastasis number and size; and set 3, Grade^anatomical^ entered the multivariate analysis instead of liver metastasis number and size, primary CRC nodal status, and extrahepatic disease.

**Table 3.**
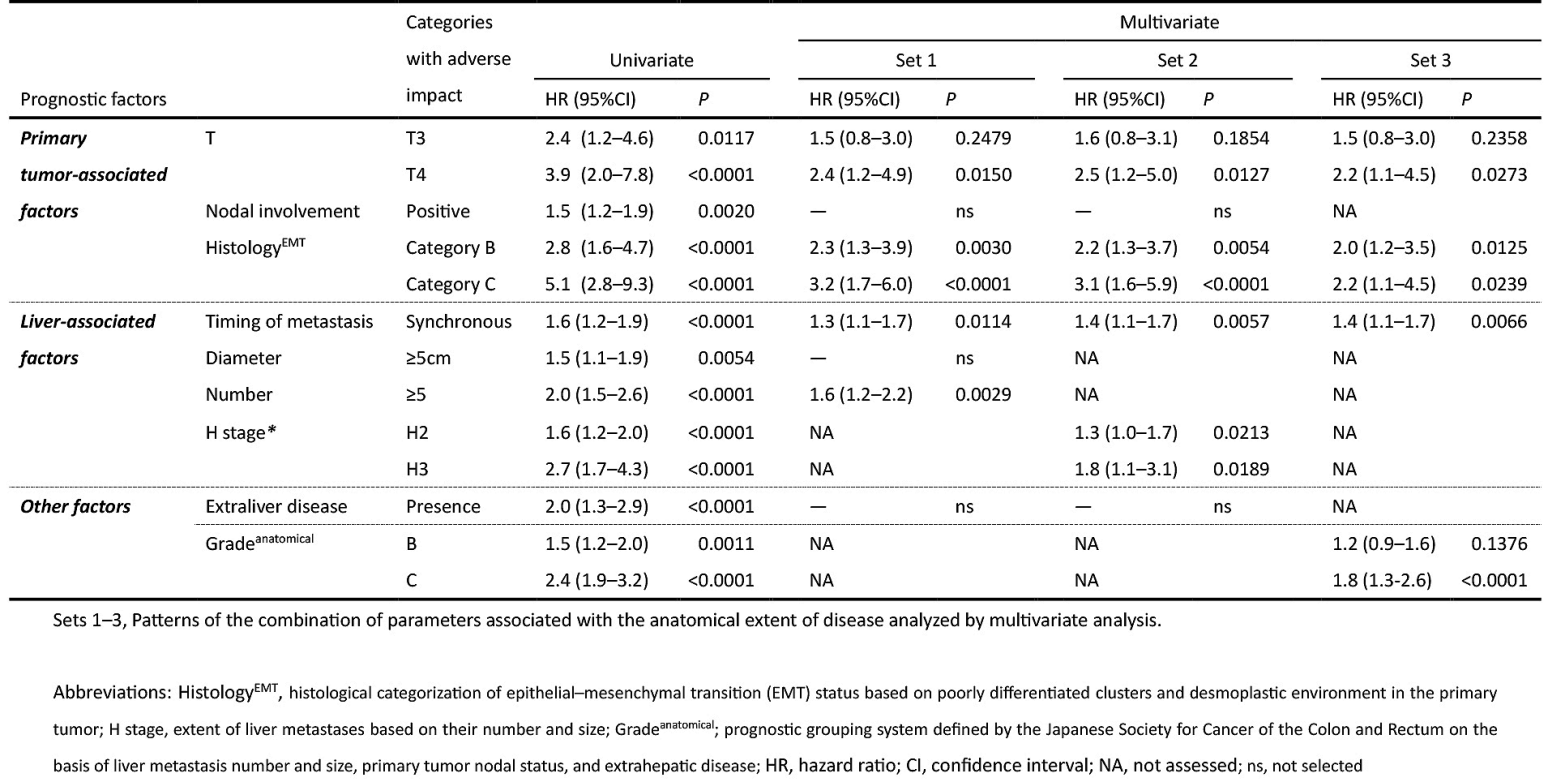
Univariate and Multivariate Analyses of Relapse-Free Survival after Hepatectomy in Patients with Colorectal Liver Metastasis.

Consequently, Histology^EMT^ was shown to significantly impact RFS independent of the anatomical extent of disease in any condition.

### Comprehensive assessment of Histology^EMT^ and Grade^anatomical^

The two-year RFS after hepatectomy was calculated for nine populations grouped by the two independent three-tiered grading systems (Histology^EMT^ and Grade^anatomical^). The survival results ranged widely: 9.9%-75.8% (Table 4). 

**Table 4.**
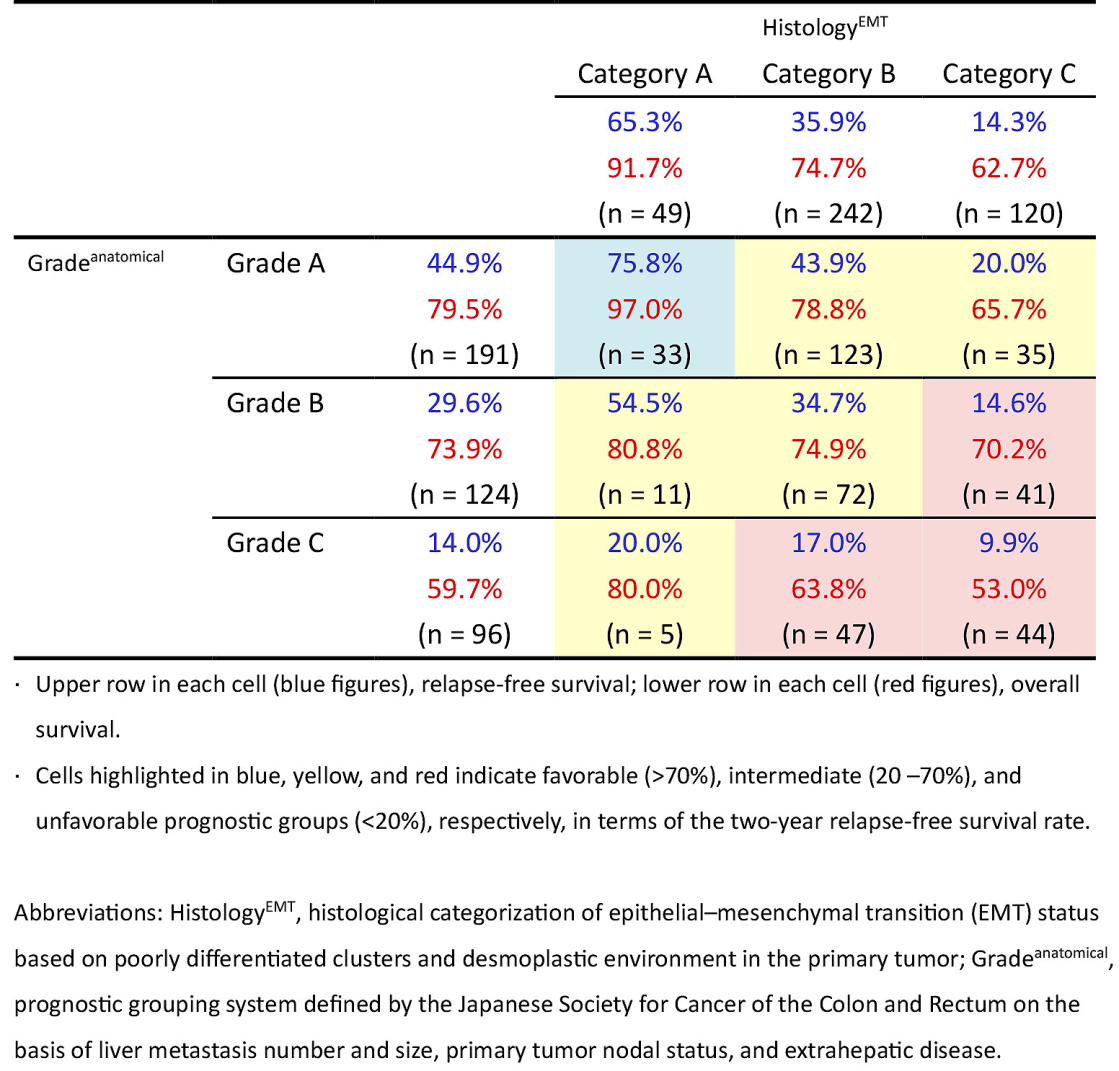
Relapse-Free and Overall Survival Rates at Two Years after Hepatectomy According to Comprehensive Assessments of Histology^EMT^ and Grade^anatomical^.

## Discussion

In the last two decades, indications for hepatectomy for CRC liver metastasis have changed considerably. Improvements in the preoperative management of hepatectomy preparations and in surgical techniques, including portal vein embolization, aggressive approaches combined with resection of adjacent major vessels, and two-stage hepatectomy, have allowed hepatobiliary surgeons to extend surgical indications for multiple or large-sized liver metastases. Additionally, even extrahepatic diseases such as lung metastases, hilar LN involvement, or peritoneal seeding are not currently regarded as contraindications of hepatectomy ^(16)^. In this recent trend of widening the indication of hepatectomy, conventional prognostic prediction systems have limited value for patient stratification for determining surgical indications ^(5)^.

Nevertheless, prognostic prediction systems with high accuracy are still valuable because they could affect clinical management in controversial areas such as delaying resection or preoperative chemotherapy ^(5)^. Japan has a prognostic grouping system for colorectal liver metastasis defined by the Japanese Society for Cancer of the Colon and Rectum (JSCCR; Grade^anatomical^), which is composed of the number and size of liver metastasis, N stage, and extrahepatic metastasis ^(14)^. Although Grade^anatomical^ has been used nationwide since 2006 when the Japanese Classification of Colorectal Carcinoma (6^th^ edition) was issued, its clinical value remains unclear; for example, its exact role in routine clinical practice has still not been defined in the Japanese guidelines ^(17)^. Similarly, international guidelines, including the National Comprehensive Cancer Network (NCCN) and European Society for Medical Oncology (ESMO) guidelines, do not use any proposed prognostic scoring systems ^(1), (2), (3), (4)^ as treatment indicators for CRC liver metastasis. This situation may indicate that there is insufficient accuracy in prognostic prediction systems using only parameters associated with the extent of disease, and novel methodologies to predict survival outcome are required.

In the present study, we focused on Histology^EMT^ comprising two EMT-associated histological factors in primary tumors: PDC and desmoplastic environment. PDC is a cancer morphology representative of dedifferentiation and mostly appears at the invasive front of the tumor. Tumor grading by quantifying PDCs has been shown to be a robust parameter for prognosis after resecting primary CRC ^(10), (18)^. In non-metastatic CRC, it was demonstrated that PDCs affect outcome independent of T and N stages and more effectively stratify the recurrence risk than the TNM tumor stage ^(10)^.

Fibrotic stroma heterogeneity at the desmoplastic front represents the variety of tumor utilization of stromal components for its development. The mechanism underlying tumor cell invasion involves complex interactions between neoplastic cells and the surrounding matrix, and recent findings have indicated that cancer develops by utilizing fibrotic stroma to modify CAF function ^(19)^. DR histological categorization based on the appearance of keloid-like collagen and myxoid stroma, which represent deviant CAF function under the influence of cancer cells, has been shown to be associated with tumor lymphocyte infiltration ^(12)^, microvascular density ^(20)^, pro-tumor extracellular matrix components (including fibronectin and tenascin-C) ^(20)^, mismatch repair status ^(21)^, and prognostic outcome in CRC patients ^(12), (20)^.

We previously found that both PDCs and DR have a prognostic value in patients with CRC liver metastasis ^(22), (23)^, and the present study clarified that these two factors are independently associated with survival results. This result is consistent with that of another previous study which showed that PDCs and DR are independent in terms of their survival impact in stage II**-**III and stage IV CRC settings ^(13)^ and are associated with the contribution of Histology^EMT^ to the effective identification of patient groups with very favorable as well as unfavorable survival results in case of CRC liver metastasis. Notably, multivariate prognostic analyses showed that Histology^EMT^ exerts a significant impact on postoperative survival after hepatectomy independent of the liver metastasis status, and AIC is the second most favorable following Grade^anatomical^, a comprehensive assessment of liver metastasis number and size, primary tumor nodal status, and extrahepatic disease. It should also be noted that Histology^EMT^ impacts postoperative survival results not only in patients with synchronous liver metastases but also in those with metachronous liver metastasis. To the best of our knowledge, this is the first study to find that CRC histology is comparable to the anatomical extent of disease in its power to stratify the prognostic outcome.

Patients with resectable liver metastases belong to a heterogeneous population in terms of oncological outcome because of the greatly diverse potential of aggressiveness that is histologically expressed in primary tumors and in the anatomical extent of disease in this group. We could resolve this issue of heterogeneity using Grade^anatomical^ and Histology^EMT^. In particular, one of the most important findings in this study was that it is possible to stratify the heterogeneous population into subgroups with wide-ranging RFS two years after hepatectomy (10%-76%) on the basis of comprehensive assessment of Histology^EMT ^and Grade^anatomical^, both of which can be determined prior to liver metastasis treatment. We believe that this approach will help resolve clinical issues originating from population heterogeneity, including effective patient selection for delaying resection and perioperative chemotherapy in patients with resectable liver metastasis who had undergone resection of the primary tumor. 

The reason why the histopathological characteristics of metastatic origin have been undervalued compared to the anatomical extent of disease is possibly owing to a lack of promising findings of conventional primary site factors. In some reports analyzing patient prognostic outcome after hepatectomy for CRC liver metastasis, poor primary tumor differentiation has had an adverse prognostic impact ^(24)^, albeit inconsistently ^(4)^. Similarly, reports of the prognostic significance of primary tumor vascular invasion are conflicting, with some reports being positive ^(25)^ and others being negative ^(4)^. We believe that our study changes this because it provides statistical evidence, including AIC and c-index, indicating that Histology^EMT^ is more powerful than these conventional primary site factors. Another reason for the underestimation of primary tumor histology in metastatic CRC is that most studies investigating prognostic outcomes after hepatectomy have been conducted by hepatobiliary surgeons who face limitations in obtaining detailed histopathological information regarding the primary tumor. We believe it would be valuable to raise awareness of the importance of prognostic stratification by adopting the latest findings in the field of gastrointestinal pathology.

In conclusion, primary tumor histology, particularly EMT-associated new prognostic parameters, should be fully considered as prognostic determinants for patients with resectable liver metastases. The information obtained by comprehensively evaluating the biological aggressiveness expressed in the primary tumor and the anatomical extent of disease could allow us to identify patients at a high risk of early relapse after hepatectomy.

## Article Information

### Conflicts of Interest

None

### Sources of Funding

This work was supported by a Grant-in-Aid for Scientific Research (C) by the Japan Society for the Promotion of Science (JSPS) KAKENHI grant number 25462075.

### Approval by Institutional Review Board (IRB)

The study protocol was approved by the Ethics Committee of the National Defense Medical College (No.2954) and the Cancer Institute Hospital (No. 2010-1056).

### Addresses of the Institutions at which this Work Was Carried Out

- National Defense Medical College (3-2 Namiki, Tokorozawa, Saitama 359-8513, Japan)
- Cancer Institute Hospital of the Japanese Foundation for Cancer Research (3-8-31, Ariake, Koto, Tokyo 135-8550, Japan)
